# Corrosion Resistance and Mechanical Properties of Cr-Rich 316 Stainless Steel Coatings Fabricated by the TIG Process Using Flux-Cored Wires

**DOI:** 10.3390/molecules29081785

**Published:** 2024-04-14

**Authors:** Peng Zhang, Huaian Jian, Lairong Yin, Jian Liu, Zhihai Cai, Yonggang Tong

**Affiliations:** 1 College of Automotive and Mechanical Engineering, Changsha University of Science and Technology, Changsha 410114, China; zphust2005@csust.edu.cn (P.Z.); zhulei@stu.csust.edu.cn (H.J.); 2 National Key Laboratory for Remanufacturing, Army Academy of Armored Forces, Beijing 100072, China; xbdliu5899@163.com (J.L.); czh@163.com (Z.C.)

**Keywords:** flux-cored wire, coatings, corrosion resistance, Cr element, Tungsten Inert Gas Welding

## Abstract

Arc welded 316 stainless steel coatings with flux-cored wires are very promising for marine service environments due to their low cost, high efficiency, and satisfactory performance, while they suffers from Cr dilution during the preparation process. Herein, based on the consideration of increasing the Cr content and ensuring the same value of the Cr/Ni equivalence ratio (Cr_eq_/Ni_eq_), 316-modified flux-cored wires, 316F (19Cr-12Ni-3Mo) and 316G (22Cr-14Ni-3Mo), were designed under the guidance of a Schaeffler diagram for the improvement of the electrochemical and mechanical properties of 316 stainless steel coatings. The designed flux-cored wires were welded into a three-layer cladding by the tungsten inert gas welding (TIG) process, and the microstructure, corrosion resistance, and mechanical properties of the claddings were investigated. The results showed that 316F and 316G consist of γ-Fe (austenite) and a small portion of δ-Fe (ferrite) as the Cr_eq_/Ni_eq_ is approximately 1.5. However, due to the higher value of the equivalent Cr content (ECC), 316G has an additional intermetallic phase (σ), which precipitates as a strengthening phase at grain boundaries, significantly increasing the tensile and yield strength of 316G but reducing its plasticity. In addition, the corrosion current density (*i_corr_*) and pitting potential (*E_b_*) for 316G are 0.20447 μA·cm^−2^ and 0.634 V, respectively, while the values for 316F are 0.32117 μA·cm^−2^ and 0.603 V, respectively, indicating that 316G has better anti-corrosion performance.

## 1. Introduction

Arc welded 316 stainless steel coatings are widely used in the remanufacturing and repairing of piping and connections for nuclear power plants due to favorable corrosion resistance and mechanical performance [1]. However, 316 stainless steel coatings exposed to seawater are subjected to long-term attack by Cl^−^ ions, which leads to severe corrosion. Pitting is a localized form of corrosion, usually the start of stress corrosion cracking as well as fatigue cracking [2,3], and is recognized as one of the most aggressive corrosion forms in stainless steel. In addition, the fracture of piping and connections will lead to further aggravation of corrosion, even failure [3]. Thus, the integrated improvement of the pitting resistance and mechanical properties of 316 stainless steel is a meaningful endeavor.

It is well known that the excellent ability to resist corrosion and mechanical performance of 316 stainless steel are related to its high Cr content [4]. Therefore, numerous research studies have been carried out on Cr in 316 stainless steel to improve its corrosion resistance. Jung et al. designed Mo-free stainless steel with high Cr concentrations, suggesting that ultra-high levels of Cr can replace Mo to improve the pitting resistance of stainless steel [5]. In recent research, the argument that the Cr/Ni equivalence ratio (Cr_eq_/Ni_eq_) has a decisive influence on the microstructure of stainless steel has received great attention [6,7,8]. Jiang et al. designed four stainless steels with high Cr_eq_/Ni_eq_ (2.65~3.19), and their results showed that the smaller the value of Cr_eq_/Ni_eq_, the more homogeneous the microstructure of the stainless steel in the range of 2.65~3.19 [6]. Hung et al. devised two gas-atomized austenitic stainless-steel powders with different Cr contents (18Cr-14Ni-2.6Mo and 22Cr-14Ni-2.5Mo) [7], which resulted in different values of Cr_eq_/Ni_eq_ (1.39 for the former and 1.66 for the latter). This study revealed that a higher value of Cr_eq_/Ni_eq_ is associated with better pitting corrosion and higher values of both tensile and yield strength but also a decline in plasticity. Similar conclusions were obtained in the study by Kim et al. [8], who indicated that Cr_eq_/Ni_eq_ greatly influences the mechanical properties of stainless steel, as it determines the value of equivalent Cr content (ECC), and in turn, it determines the formation of the σ phase in austenitic stainless steel weldments. The σ phase, a hard and brittle intermetallic phase, might reduce the toughness and ductility of stainless steel [9,10]. However, given that the presence of the σ phase is often accompanied by high tensile and yield strengths, it can also be regarded as a strengthening phase.

Currently, research on 316-modified materials focuses on the following areas: stainless steel powders applied to the laser powder bed fusion (LPBF) process [11,12], stainless steel ingots applied to vacuum induction melting (VIM) [5,13,14,15], and stainless steel welding wires applied to cladding processes [16,17,18]. Stainless steel powder has remarkable processing capabilities and is widely used in manufacturing stainless steel components, precision 3D printing, and coating metal surfaces [19,20]. Nonetheless, its exorbitant expense renders it unsuitable for large-scale part repairs [20,21]. Stainless steel materials used in melting and casting are typically supplied in ingots, which are both cost effective and efficient, making them widely used to manufacture a range of stainless steel parts [19,22]. Nevertheless, applying melting and casting techniques for repairing old parts is not feasible. Therefore, stainless steel wire is widely used in the remanufacturing and repairing of broken pipes and connections due to its good formability, low cost, and high efficiency [23]. Solid wire is a good choice for its satisfactory welding manufacturability. However, as the Cr content increases, solid core wire shows an increase in tensile strength and a decrease in plasticity, making the preparation process more difficult. Under such circumstances, flux-cored wires constitute the best choice owing to the ease of regulating the chemical composition. In addition, the welding wire is always accompanied by penetration of matrix elements during the cladding process [24,25,26], which leads to dilution of the Cr element in the cladding layer. An increase in the Cr content of flux-cored wires would be able to compensate for Cr dilution during cladding, which in turn would decrease the Cr-depleted area in the cladding layer.

In this work, the 316-modified flux-cored wire, 316F (19Cr-12Ni-3Mo), was designed with Cr_eq_/Ni_eq_ of 1.5 under the guidance of a Schaeffler diagram. Based on the consideration of increasing the Cr content and ensuring the same value of Cr_eq_/Ni_eq_, another flux-cored wire, 316G (22Cr-14Ni-3Mo), was designed and applied in the TIG process. The microstructure, corrosion resistance, and mechanical properties of 316F and 316G were investigated. The results show that the microstructures of 316G and 316F are approximately the same, while the higher value of ECC results in an additional intermetallic phase σ in 316G, which significantly increases its tensile and yield strength but reduces its plasticity. On the other hand, the increased Cr content of 316G also improves its PREN, giving a more stable passivation film and better electrochemical performance during corrosion. This work provides a new idea for comprehensively improving the pitting resistance and mechanical properties of arc welded 316 stainless steel coatings.

## 2. Results and Discussion

### 2.1. Microstructure and Physical Analysis

**Figure 1:**
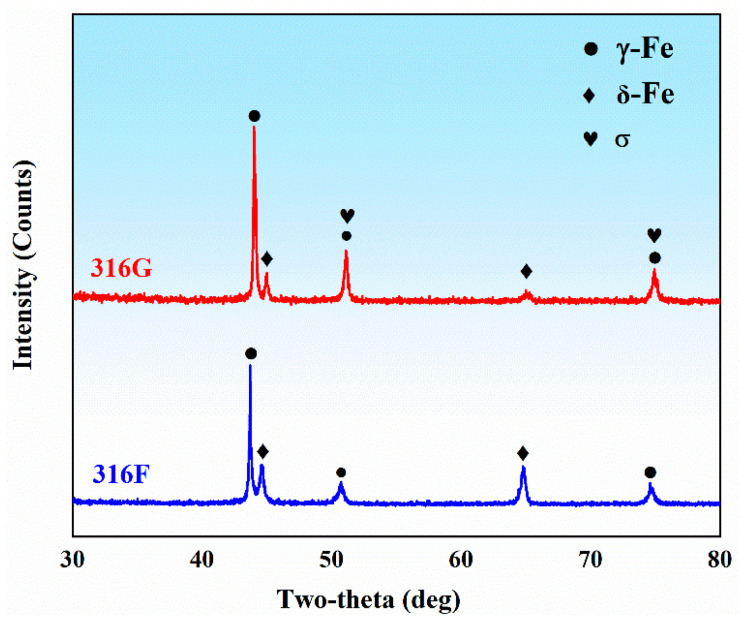
X-ray diffractograms of 316F and 316G.

**Figure 2:**
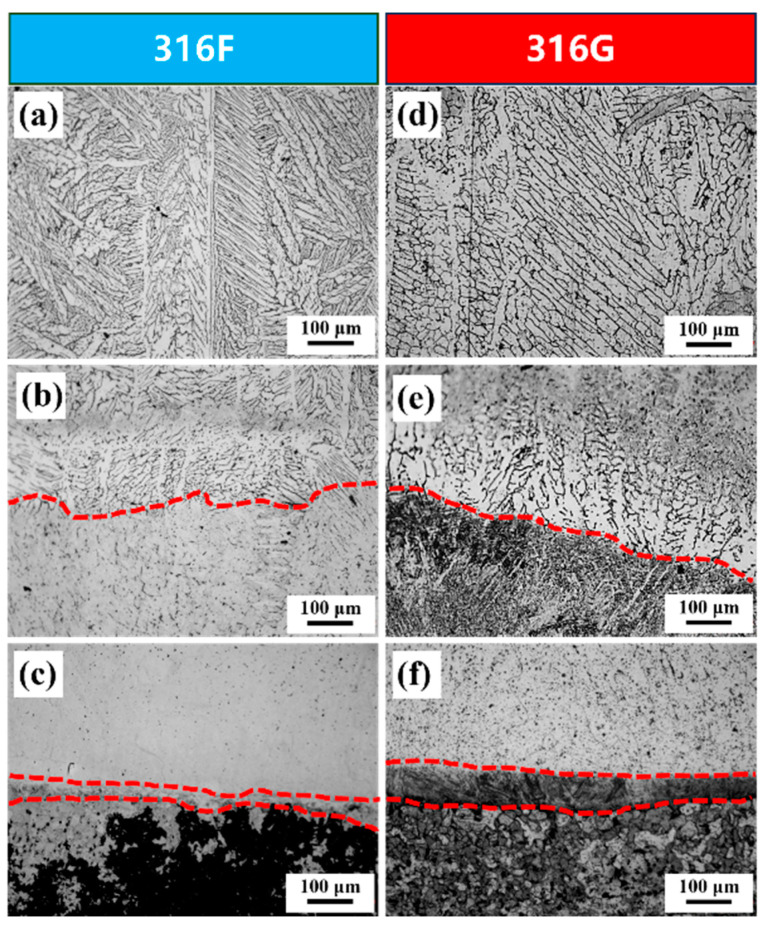
Metallographic structures of 316F and 316G. (**a**) The top layer of 316F. (**b**) Joint area of the first layer and the second layer of 316F. (**c**) Joint area of the matrix and the first layer of 316F. (**d**) The top layer of 316G. (**e**) Joint area of the first layer and the second layer of 316G. (**f**) Joint area of the matrix and the first layer of 316G.

The XRD spectra for 316F and 316G are presented in Figure 1. In this figure, γ-Fe and δ-Fe peaks of the same type were observed simultaneously in the two materials. However, the intensity of some peaks showed variability between the different materials, which may have been related to the different values of Cr_eq_ and Ni_eq_, as they determine the solidification pattern during the preparation of the coatings and the final microstructure. The microstructure of 316F and 316G was composed of austenite and a small amount of ferrite, as shown in Figure 2, which was consistent with the results of XRD analysis. The δ-ferrite (δ-Fe) was predominantly dendritic and distributed in the γ-austenite matrix (γ-Fe) in 316F, whereas it was dispersed δ-Fe in 316G, as shown in Figure 2d,e.

**Figure 3:**
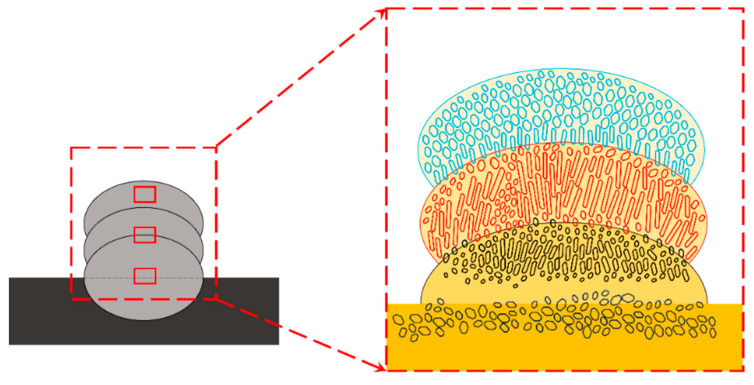
The distribution and growth of the grains.

The distribution and growth of the grains are shown schematically in Figure 3. In addition to their similar microstructure, the growth directions and growth patterns of the two materials showed good agreement. The grains grew in the direction of heat transfer and showed an overall tendency to increase in size, which was due to the progressive deterioration of heat dissipation conditions during the cladding process [27]. On the other hand, large columnar and equiaxed crystals were distributed in the center of the clad layer, while small grains were distributed at the bottom and top. This could be attributed to the interlayer cooling during the preparation of the cladding area [28]. 

**Figure 4:**
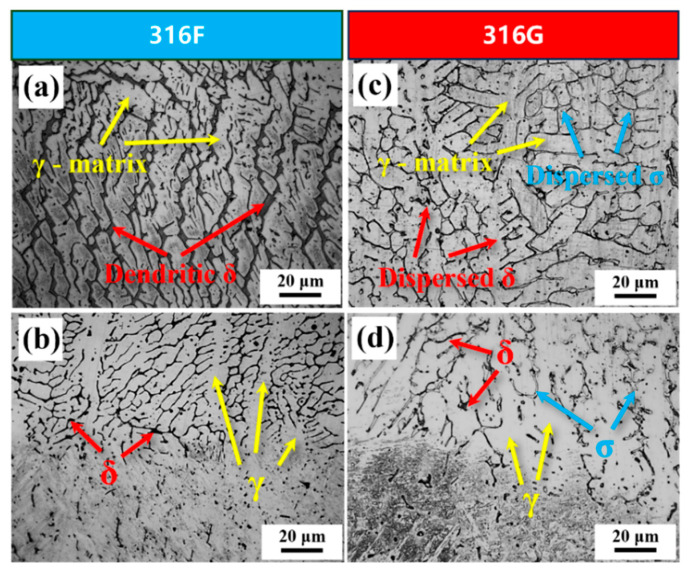
(**a**,**b**) γ-Fe, δ-Fe observed in 316F. (**c**,**d**) γ-Fe, δ-Fe, and the σ phase observed in 316G.

It is worth noting that, although not mentioned in Equation (12), the σ phase appeared in 316G. By contrast, it was not observed in 316F, as illustrated in Figure 4. The σ phase is routinely found and given great attention in a large number of studies on stainless steel, particularly in those with high contents of elements such as Cr, Ni, Mo, etc. [29,30,31].

### 2.2. Electrochemical Corrosion Behavior

#### 2.2.1. Electrochemical Impedance

**Figure 5:**
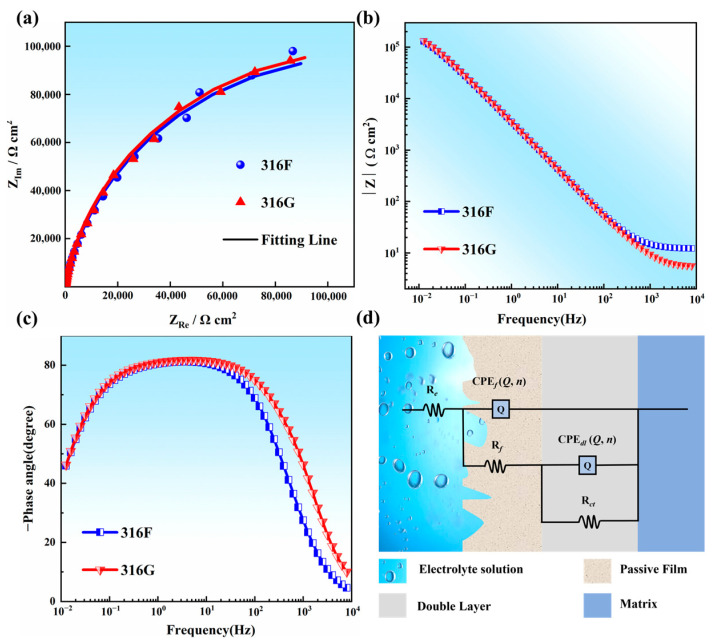
EIS spectra. (**a**) Nyquist plots of 316F and 316G. (**b**,**c**) Bode plots of 316F and 316G. (**d**) Equivalent circuit for EIS test.

Figure 5 shows the EIS data as Nyquist and Bode plots for 316F and 316G. The Nyquist curves of all the samples showed an arc, indicating a capacitive metal/electrolyte interface [32,33], while the impedance moduli were almost identical, as shown in Figure 5a,b.

The frequency-phase distribution of all samples is presented in Figure 5c. The curves of 316F and 316G showed a plateau (−70~−80°) over a wide frequency range (10^−1^~10^2^ Hz), suggesting the satisfactory capacitive behavior of the electrical double layer formed on the alloy surface. Note that the surface of a stainless steel passivation film is two-layered, with a thick and loosely textured outer layer and a thin but compact inner layer [34,35]. Hence, the metal/electrolyte interface with a double-layer passive film is expressed by equivalent electrical circuits with two constant phase elements, as depicted in Figure 5d. In this figure, *R_e_*, *R_f_*, and *R_ct_* reflect the resistance of the electrolyte, the passive film, and the charge transfer, respectively. In addition, *CPE_f_* and *CPE_dl_* describe the electrochemical response of the passive film and the electrical double layer at the metal/electrolyte interface, respectively.

Table 1 presents the fitting parameters of all EIS data. Parameter (*γ*) and exponent (*n*) were applied to characterize *CPE_f_* and *CPE_dl_*. All samples showed an increasing trend from *R_e_* to *R_f_*, indicating that all samples were resistant to corrosion in 3.5 wt.% NaCl solution.

To quantify the electrochemical properties of the passivation film, Equations (1) and (2) were adopted to estimate the capacitance of the passivation film (*C_pass_*) and the thickness of the passivation film (*δ*), respectively, based on the work of Hirschorn et al. [36].

(1) $$C_{pass}=\gamma^{1/n}R^{(1-n)/n}$$

(2) $$\delta =\varepsilon \varepsilon_{0}/C_{pass}$$

where *ε* is the dielectric constant and *ε*_0_ = 8.8542 × 10^−14^ F/cm is the permittivity under vacuum. Note that, in this work, the corrosive medium was 3.5 wt.% NaCl and *ε* = 78 [37].

As shown in Table 2, the *C_pass_* and *δ* values indicated that the passivation film of 316G was thicker than that of 316F in 3.5 wt.% NaCl solution, and a thicker passivation film generally represents better corrosion resistance as it decreases the penetration of Cl^−^ ions.

#### 2.2.2. Potentiodynamic Polarization

**Figure 6:**
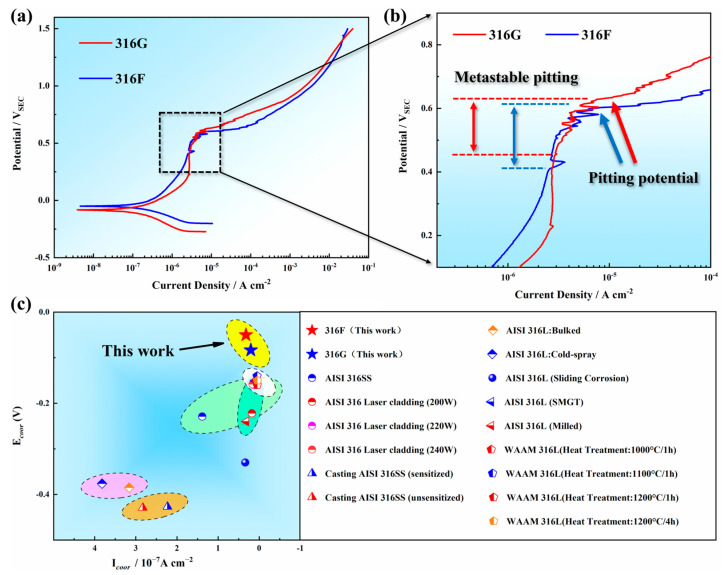
(**a**) Potentiodynamic polarization curves of 316F and 316G. (**b**) Magnification of the selected region in (**a**). (**c**) Comparison of the electrochemical properties of austenitic stainless steels in several existing studies with the present work.

The pitting corrosion resistance and the electrochemical process of all specimens were comparatively investigated by potentiodynamics, and the polarization curves of all samples are exhibited in Figure 6a,b. The anode region of 316G exhibited a nearly constant current density within the working electrode potential range of 0.2~0.5 V_SCE_. While in the same potential range, the current density in the anodic region of 316F was characterized by a slow increase from 1.28 × 10^−6^ A·cm^−2^ to 3.01 × 10^−6^ A·cm^−2^. Based on the variation of anodic current density, it can be inferred that 316G exhibited greater stability within the above potential range.

Metastable pitting [38,39] occurred at the end of the passive region for both 316F and 316G. The value of the pitting potential (*E_b_*) of 316G was 0.634V, which was slightly higher than that of 316F, as shown in Figure 6b. This could be attributed to the higher value of the pitting resistance equivalent number (PREN) [40] of 316G, as it is an important factor in measuring the resistance of stainless steel to pitting corrosion. The value of PREN can be calculated by Equation (3). The formula indicates that the pitting corrosion resistance of stainless steel is significantly influenced by elements such as Cr, Mo, and N. Therefore, it can be inferred that the higher pitting potential of 316G in this work was due to its higher Cr content.

(3) $$\mathrm{PREN}=\mathrm{Cr}+3.3\mathrm{Mo}+16N\;\left(\%\right)$$

The magnitude of the corrosion rate can be reflected by the corrosion current density (*i_co__rr_*) and the corrosion potential (*E_co__rr_*). The two parameters (*i_co__rr_* and *E_co__rr_*) can be extrapolated by Tafel extrapolation, and they were compared with those of several austenitic stainless steels, such as 316 and 316L stainless steels, for different processes and conditions, or their modified stainless steels in the existing research, as shown in Figure 6c [1,41,42,43,44,45]. The comparison showed that the stainless steel designed in this work had a lower value of *i_co__rr_* and a higher value of *E_co__rr_*, indicating better electrochemical performance.

The values of *i_co__rr_*, *E_co__rr_*, and *E_b_* for all specimens are presented in Table 3. Both values of *i_co__rr_* and *E_co__rr_* were in the order of 316F > 316G. This demonstrated that 316G boasted the lowest corrosion rate. In addition, the higher value of *E_b_* indicated better pitting resistance for 316G. The conclusion that can be drawn from the above discussion is that 316G possessed the best comprehensive corrosion resistance in this work.

#### 2.2.3. Corrosion Morphology and Corrosion Mechanism

**Figure 7:**
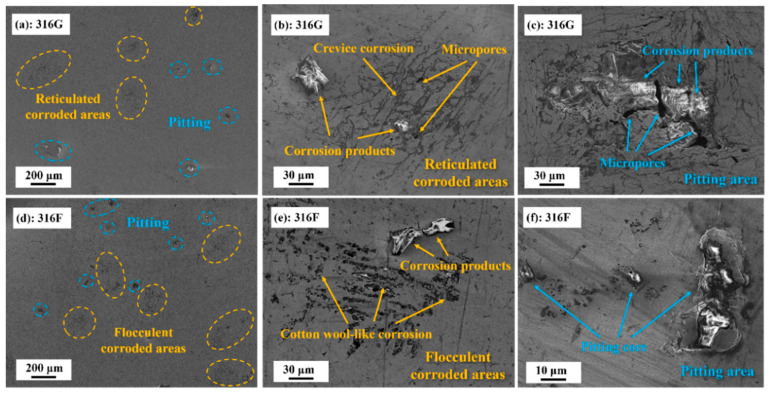
(**a**) Corrosion morphology with pitting and reticulated corrosion areas of 316G. (**b**) The reticulated corrosion areas of 316G. (**c**) Pitting area on the surface of 316G. (**d**) Corrosion morphology with pitting and reticulated corrosion areas of 316F. (**e**) The flocculent corrosion areas of 316F. (**f**) Pitting area on the surface of 316F.

The corrosion morphologies of all specimens are presented in Figure 7. Large areas of minor corrosion (reticulated corrosion zones) and pitting were scattered over the surface of 316G, as shown in Figure 7a. To illustrate the corrosion process of 316G, different degrees of surface corrosion are shown in Figure 7b,c. The reticulated corrosion zones consisted of numerous corrosion crevices of varying depths and a small number of micropores and corrosion products (shown in Figure 7b). As corrosion progressed, the crevices and micropores on the surface of the reticulated corrosion area expanded and fused, eventually forming a severe pitting zone containing many deep micropores and corrosion products (shown in Figure 7c). In the case of 316F, the minor corrosion zones and pitting were more intensive than in 316G, as shown in Figure 7d,e. In addition, the minor corrosion zones of 316F were flocculent, according to Figure 7f, which were wider and deeper than the corrosion crevices in the reticulated corrosion area of 316G. The difference in corrosion resistance between 316F and 316G was reflected to some extent in the corrosion surface, indicating that deeper research and discussion must revolve around the corrosion principles of stainless steel in 3.5 wt.% NaCl.

**Figure 8:**
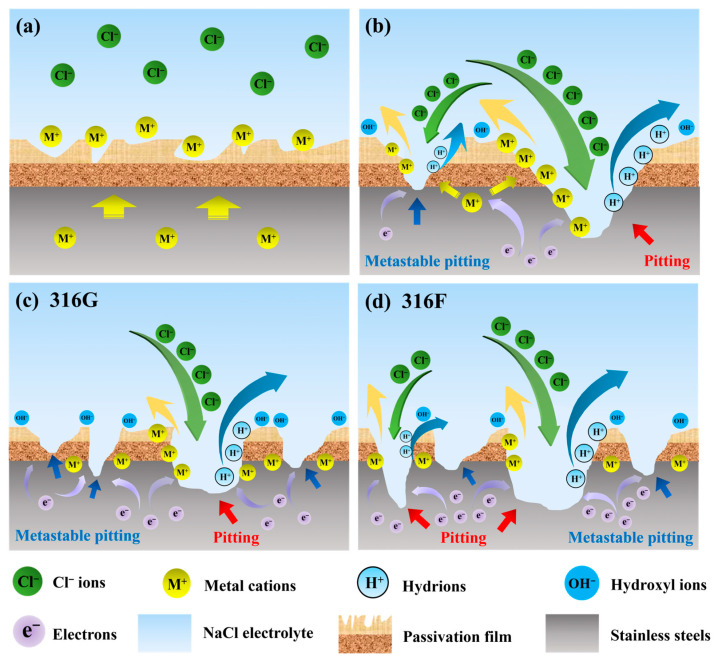
(**a**) Passivation films on stainless steel surfaces in 3.5 wt.% NaCl electrolyte. (**b**) Mechanisms of pitting and metastable pitting on stainless steel surfaces. (**c**) Corrosion of passivation film surface of 316G. (**d**) Corrosion of passivation film surface of 316F.

The passivated stainless steel surfaces in 3.5 wt.% solution are illustrated in Figure 8a. Substantial amounts of free Cl^−^ ions are dispersed in the solution, while the metal cation M^+^ (mainly Cr^3+^, Fe^2+^, Ni^2+^) is mainly distributed near the passivation film on the stainless steel surface. The Cl^−^ ions and the metal cation M^+^ are stable at the beginning of passivation. As the corrosion potential increases, many electrons in the stainless steel migrate toward the working electrode (i.e., the cathode of stainless steel), causing its negative potential [46]. By contrast, the passivation film shows a positive potential, leading to many Cl^−^ ions arriving and launching an attack [47,48]. The passivation film is continuously eroded due to the enrichment of Cl^−^ ions, and metal elements such as Cr, Fe, Ni, etc. on the passivation film enter the solution in the ionic state. Meanwhile, active metal atoms lose their electrons and enter the passivation film, which is called the reparation of the passivation film [47]. The reactions occurring at the passivation film during this process are shown in Equations (4)–(8).

(4) $$\mathrm{Cr}\to {\mathrm{Cr}}^{3+}{+3e}^{-}$$

(5) $$\mathrm{Fe}\to {\mathrm{Fe}}^{2+}{+2e}^{-}$$

(6) $$\mathrm{Ni}\to {\mathrm{Ni}}^{2+}{+2e}^{-}$$

(7) $$O_{2}{+2H}_{2}{O+4e}^{-}\to {4(\mathrm{OH})}^{-}$$

(8) $$M^{2+}{+H}_{2}O\to {M(\mathrm{OH})}^{+}{+H}^{+}$$

The “demand” of the passivation film for metal cations depends on the corrosion potential, while the “supply capability” of the stainless steel for metal cations depends on the elemental content. Given that the chemical element content of stainless steel is constant, the relationship between “demand” and “supply capability” is determined by the magnitude of the corrosion potential. In this work, as the corrosion potential increased from 0 V to 1.5 V, the relationship between “demand” and “supply capability” went through the following three stages:

(1) When the “demand” is less than the “supply capability,” the potential difference between the cathode and the anode of stainless steel is stable, thus the corrosion current density remains almost constant. In the present work, this result was validated in the polarization curves for corrosion potentials of 0.3~0.43 V, as shown in Figure 6b.
(2) The passivation film becomes unstable when the “demand” approaches the “supply capability,” as illustrated on the left half of Figure 8b. The voltage between two poles of stainless steel increases and drops instantaneously as the passivation film is penetrated or repaired, which results in a steep increase in the magnitude of the corrosion current density. This phenomenon is described as metastable pitting, which corresponds to the polarization curves in Figure 6b with corrosion potentials of 0.5~0.62 V.
(3) When the “demand” exceeds the “supply capability,” the passivation film will be completely punctured, and the stainless steel enters the pitting phase, as shown in the right half of Figure 8b. Thenceforth, even a small increase in corrosion potential will bring about a surge in corrosion current density, which was reflected in the polarization curves after 0.62 V in the present work (shown in Figure 6a).

Theoretically, the “supply capacity” can be interpreted as the ability of stainless steel to maintain a constant voltage at the cathode and anode and is ultimately related to the ability of its elements to gain or lose electrons. It can be seen from Equations (4)–(6) that Cr must lose 3 electrons to change from an atom to an ion, suggesting a greater ability to gain or lose electrons than Fe and Ni. In addition, Cr is the main element that constitutes and potentiates the passivation film of stainless steel. As a result, Cr plays the most important role from the formation of the passivation film to its breakdown.

Consequently, although both metastable pitting and steady pitting occurred in the corrosion process of 316F and 316G (shown in Figure 8c,d), the higher Cr content brought 316G smaller corrosion zones, slighter corrosion pits, and a higher value of pitting potential than 316F (shown in Figure 6b and Figure 7).

### 2.3. Mechanical Properties

**Figure 9:**
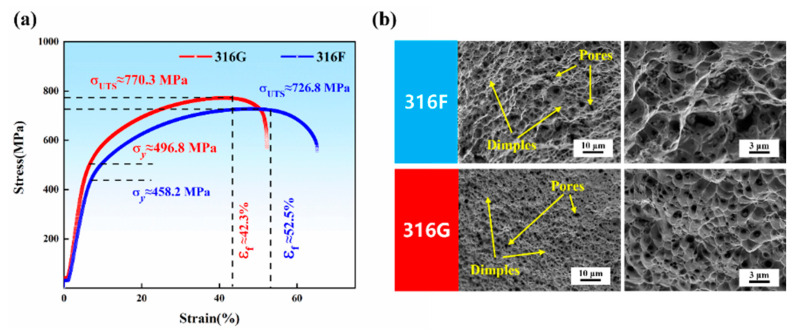
(**a**) Tensile strain–stress curves. (**b**) Fracture surface morphologies of 316F and 316G.

Stress–strain curves for all specimens are shown in Figure 9a to investigate the tensile properties of 316F and 316G. According to the curves, all specimens underwent elastic deformation, plastic deformation, and fracture stages. The tensile strength and yield strengths of 316G were 770.3 MPa and 496.8 MPa, respectively, while the values of 316F were 726.8 MPa and 458.2 MPa, respectively. The result that 316G had higher strength could be attributed to the formation of the σ-Fe phase near grain boundaries, which provided a better strengthening effect than the δ-Fe phase [44]. However, 316G showed poorer plasticity than 316F, as more σ-Fe phase was produced. σ-Fe phase formation in austenitic stainless steel can be predicted through ECC [8], and the values of ECC_316F_ (22.893) and ECC_316G_ (25.361) were calculated using Equation (9).

(9) $$\begin{array}{l} \mathrm{ECC}=\mathrm{Cr}+0.31\mathrm{Mn}+1.76\mathrm{Mo}+0.97W+2.02V+1.58\mathrm{Si}\\ +2.44\mathrm{Ti}+1.7\mathrm{Nb}+1.22\mathrm{Ta}-0.266\mathrm{Ni}-0.177\mathrm{Co}(\%) \end{array}$$

The fracture surface morphologies of 316F and 316G are presented in Figure 9b. Numerous dimples were observed in the tensile fractures of all specimens, indicating that the failure mechanism for 316F and 316G was a ductile fracture. In addition, 316F had significantly larger dimples than 316G, which corresponded precisely with its good plasticity.

## 3. Experiment

### 3.1. Material Modification

In this study, 316F (19Cr-12Ni-3Mo) with Cr_eq_/Ni_eq_ of 1.5 was designed by introducing a small amount of ferrite into 316 stainless steel under the guidance of a Schaeffler diagram [7]. This is advantageous for stainless steel because pure austenite is highly sensitive to solidification cracking, and the addition of small amounts of ferrite has proven effective in reducing the susceptibility of the cladding metal to thermal cracking [49]. Further, 316G (22Cr-14Ni-3Mo) was designed based on the consideration of increasing the Cr content and ensuring the same Cr_eq_/Ni_eq_. The chemical components of 316F, 316G, and conventional 316 stainless steel are provided in Table 4.

With the chemical composition of 316F and 316G, the values of Cr_eq_ and Ni_eq_ could be calculated by the Schaeffler formula (Equations (10) and (11)) [8], which are summarized in Table 5.

(10) $${\mathrm{Cr}}_{\mathrm{eq}}=\mathrm{Cr}+\mathrm{Mo}+1.5\mathrm{Si}+0.5\mathrm{Nb}(\%)$$

(11) $${\mathrm{Ni}}_{\mathrm{eq}}=\mathrm{Ni}+30C+0.5\mathrm{Mn}(\%)$$

The Cr_eq_/Ni_eq_ value of 1.5 suggested that the solidification mode of 316F and 316G was FA [8], i.e., it followed the solidification process as shown in Equation (12).

(12) L→(L+δ)→(L+δ+γ)→(γ+δ)

where L is the liquid, δ is delta-ferrite, and γ is austenite. With Equation (12), δ ferrite is generated first by liquid phase transformation, followed by γ austenite. This mode of solidification ensures that the microstructure of the cladding layer consists of mostly austenite and a small portion of ferrite. 

**Figure 10:**
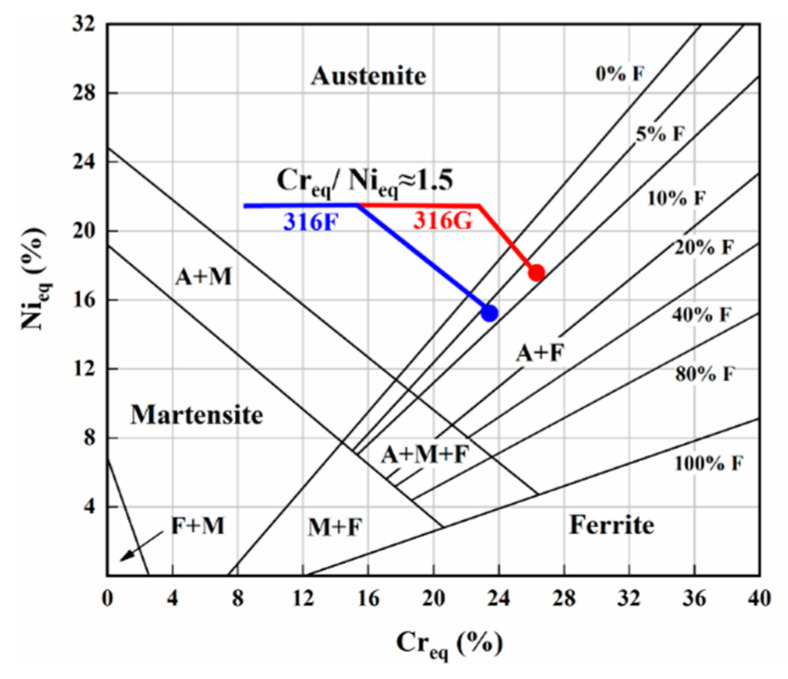
Schaeffler diagram for 316F and 316G.

The Schaeffler diagram for 316F and 316G was obtained from Table 5 and is shown in Figure 10. As estimated in this figure, the expected microstructure of 316F and 316G was predominantly austenitic (>90%), with small amounts of ferrite (5~10%). Note that as many of the key elements (such as Cr, Ni, and Mo) were depleted as the matrix elements were penetrated during preparation of the coatings, the real microstructure might differ from the expected one. However, the depletion was minimal, as the specimens used for testing in this work were sampled in the third layer of the coatings.

### 3.2. Sample Preparation

**Figure 11:**
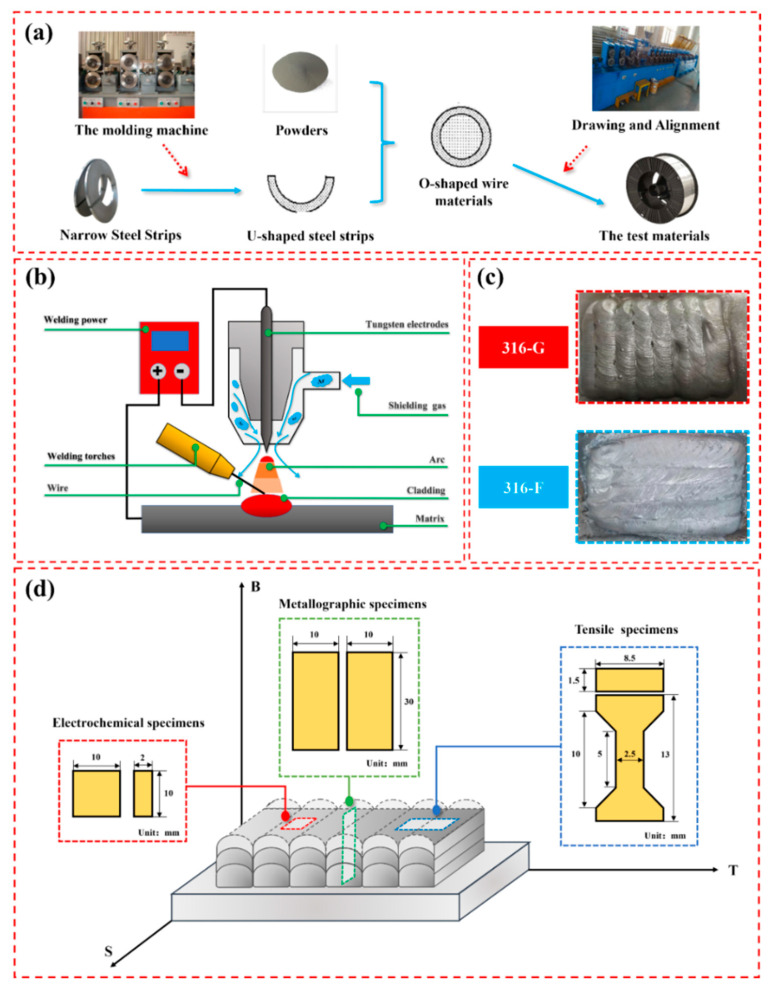
(**a**) Preparation of test materials. (**b**) TIG principle of operation. (**c**) Surface of the cladding. (**d**) Sampling locations of all specimens.

The preparation process of 316F and 316G is shown in Figure 11a. The alloy wire forming and manufacturing equipment was obtained from Changsha Jilin Machinery Equipment Co. (Changsha, China). The strip used to prepare flux-cored wires was annealed 430 stainless steel with a size of 11 mm × 0.3 mm, which was cleaned before entering the forming machine. The powder used to prepare flux-cored wires was 80 mesh, purchased from Jinzhou Sihai Metal Company Limited (Jinzhou, China) and mechanically mixed and dried before use. After preparation, 316G and 316F were welded into three-layer cladding by an Inverter Pulse Tungsten Inert Gas (TIG) Welding System chased from Shandong Autai Electric Co. (Jinan, China)with the same cladding process parameters. The Q345 steel with dimensions of 100 mm × 200 mm × 10 mm was selected as the substrate. The experimental sketch of TIG and the cladding surface of all materials are shown in Figure 11b and Figure 11c, respectively. The cladding process parameters are summarized in Table 6.

### 3.3. Microstructure Characterization

All specimens were taken from the top layer of the melt, as shown in Figure 2d. The metallographic specimens were polished successively with 400#, 800#, 1500#, and 2000# metallographic sandpaper. All specimens were polished to a mirror finish by a P-1000 polisher and then ultrasonically cleaned and dried. The polished metallographic specimens were etched with FeCl_3_: HCl: C_2_H_5_OH solution in a volume ratio of 5:2:99 (volume ratio) for 15 s. The metallographic structure was observed using an IE500M optical microscope manufactured by Ningbo Sunyu Instruments Co. (Ningbo, China). The phase compositions of 316F and 316G were examined using an X-ray diffractometer utilizing Cu-Kα rays as the radiation source at an operational voltage of 400 kV and a current of 400 mA. Data were collected for 2θ = 30° to 80° at a scan rate of 5°/min. The experimental data were then analyzed by MDI-Jade 6.0 to determine the phase composition of the alloy.

### 3.4. Electrochemical Test

Electrochemical tests were carried out in 3.5 wt.% NaCl using the Solartron 1470E electrochemical workstation obtained from Solartron Analytical (Farnborough, UK). Electrochemical samples with the size of 10 mm × 10 mm × 2 mm were used as the working electrode. A platinum sheet electrode was adopted as the auxiliary electrode, and a saturated potassium chloride/glyceryl electrode (SCE) was utilized as the reference electrode. Constant potential polarization (−0.8 V_SCE_ for 10 min), open circuit voltage (OCP) test (1 h), electrochemical impedance spectroscopy (EIS) testing (10^−2^~10^6^ Hz with an amplitude of 10 mV), and dynamic potential scanning polarization testing (−0.25~1.5 V_SCE_) were performed in sequence. All electrochemical samples were taken from the top layer of the melt and ground to 2000# grade, and the electrochemical tests were carried out at room temperature.

### 3.5. Mechanical Properties Test

Tensile tests were performed using a CMT5105GL electronic universal tensile machine manufactured by Zhuhai SUST Electrical Equipment Co., Ltd. (Zhuhai, China) with a strain rate of 5 × 10^−3^ s^−1^ at room temperature. Three tensile tests were performed for each sample and the results were averaged to ensure accuracy.

## 4. Conclusions

In this work, two 316-modified flux-cored wires, 316F (19Cr-12Ni-3Mo) and 316G (22Cr-14Ni-3Mo), were prepared and applied in the tungsten inert gas welding process. The Cr_eq_/Ni_eq_ value of 316F and 316G was set as 1.5 to obtain an adequate microstructure (predominantly austenite and a small portion of ferrite), and the main conclusions are as follows:

(1) 316G exhibits better corrosion resistance than 316F based on conducted potentiodynamic polarization testing and corrosion morphology observation, which is mainly manifested in lower corrosion current density (*i_corr_*), higher pitting potential (*E_b_*), and milder corrosion morphology.
(2) The passivation process of stainless steel is essentially a change in the “supply” and “demand” of metal cations. The element Cr shows excellent ability to gain and lose electrons during corrosion, which is conducive to protecting the passivation film from being punctured, thus improving the corrosion resistance of stainless steel.
(3) 316F exhibits a tensile strength of 726.8 MPa and a yield strength of 458.2 MPa, along with an exceptionally high elongation of 52.5%. However, as the EEC is boosted with elevated Cr, the hard phase σ-Fe is formed as a reinforcing phase in 316G, contributing to its higher tensile (770.3 MPa) and yield (496.8 MPa) strengths while reducing its plasticity (42.3%).

## Figures and Tables

**Table 1:** Fitting parameters for EIS.

Specimen	*R_e_* (Ω·cm^2^)	*R_f_* (Ω·cm^2^)	*CPE_f_*		*R_ct_* (Ω·cm^2^)	*CPE_dl_*		Chi-Square
			*γ* (Ω^−1^·cm^−2^·s^−n^)	*n*		*γ * (Ω^−1^·cm^−2^·s^−n^)	*n*	
316F	12.14	35.08	2.495 × 10^−5^	0.9806	2.31 × 10^5^	2.93 × 10^−5^	0.8228	0.000331
316G	5.46	44.03	2.761 × 10^−5^	0.9720	2.33 × 10^5^	2.48 × 10^−5^	0.8322	0.000617

**Table 2:** Values of *C_pass_* and *δ* for all samples.

Specimen	316F	316G
*C_pass_*/F·cm^−2^	4.43 × 10^−5^	3.49 × 10^−5^
*δ*/nm	1.559	1.985

**Table 3:** Values of $i_{corr}$, $E_{corr}$, $E_{b}$, and PREN for all specimens.

Specimen	*i_corr_*/μA·cm^−2^	*E_corr_*/V	*E_b_*/V	PREN
316F	0.32117	−0.0499	0.603	28.9%
316G	0.20447	−0.0833	0.634	31.9%

**Table 4:** The chemical composition of all materials (%).

Materials	C	Si	Mn	Cr	Ni	Mo	P	S	Nb	Fe
316G	0.08	0.75	2	22	14	3	≤0.2	≤0.25	0	Bal.
316F	0.08	0.75	2	19	12	3	≤0.2	≤0.25	0	Bal.
316	≤0.08	≤1	≤2	16~18	10~14	2~3	≤0.045	≤0.03	0	Bal.

**Table 5:** Values of Cr_eq,_ Ni_eq,_ and Cr_eq_/Ni_eq_ for all samples.

Specimen	Cr_eq_	Ni_eq_	Cr_eq_/Ni_eq_
316F	23.13	15.4	1.5019
316G	26.13	17.4	1.5017

**Table 6:** The cladding process parameters.

Cladding Manner	Cladding Current /A	Cladding Voltage /V	Cladding Rate /mm·s^−1^	Shielding Gas Rate /L·min^−1^
TIG	200	18	2.5	15

## Data Availability

Data are contained within the article.
